# *Environmental Health*: the first five years

**DOI:** 10.1186/1476-069X-6-27

**Published:** 2007-09-11

**Authors:** Philippe Grandjean, David Ozonoff

**Affiliations:** 1 Department of Environmental Medicine, University of Southern Denmark, Odense, Denmark; 2 Department of Environmental Health, Harvard School of Public Health, 401 Park Drive-3E, Boston, MA, USA; 3 Department of Environmental Health, Boston University School of Public Health, 715 Albany Street, Boston, MA, USA

## 

*Environmental Health *is now firmly established as a major venue for publishing in the field of environmental health. While remaining selective in our acceptances – of the 217 manuscripts that we have processed by June 2007, 115 (53%) were accepted – the number of manuscripts continues to grow from year to year. Last year we published 33 articles (of 64 submitted) and the number of submissions by June this year has already reached 40. The journal has now been in existence for five years, so the time seems ripe for us to assess the health of our journal and the opportunities offered by open access publication on the Internet.

## Digital technology

Published articles are one of the main products of research. Digital technology is revolutionizing the reproduction, distribution and control of scientific publication while providing new and important opportunities for science. It also allows wider and more rapid diffusion of knowledge with lower barriers to access, stimulating further research and applications of new information. The Internet facilitates access to journals that would otherwise require a visit to many different libraries. The majority of scientific journals are now available online, and most of the 2.5 million scientific articles published per year can be accessed away from libraries that carry the print versions.

For this reason, "pigeon-holing" of research areas has become less important for the researcher to reach the intended audience. As the actual medium in which the scientific article appears becomes less crucial, the choice of one topic-related journal over another among the almost 25,000 peer-reviewed journals worldwide, now includes the criterion of accessibility.

## Open access

Open access is both a colloquial term for no cost access to scholarly publications and a specific set of principles governing publication and the kind of permissions needed for its further use. As part of the suite of journals published by BioMed Central *Environmental Health *adheres strictly to the Open Access charter[1]:

1. The article is universally and freely accessible via the Internet, in an easily readable format and deposited immediately upon publication, without embargo, in an agreed format – current preference is XML [Extensible Mark-up Language] with a declared DTD [Document Type Definition] – in at least one widely and internationally recognized open access repository (such as PubMed Central).

2.The author(s) or copyright owner(s) irrevocably grant(s) to any third party, in advance and in perpetuity, the right to use, reproduce or disseminate the research article in its entirety or in part, in any format or medium, provided that no substantive errors are introduced in the process, proper attribution of authorship and correct citation details are given, and that the bibliographic details are not changed. If the article is reproduced or disseminated in part, this must be clearly and unequivocally indicated.

Whether open access or subscription-based, there are usually costs associated with publication, costs borne by the author (usually about $2,000; at current exchange rates it is about $1500 for *Environmental Health*), or by the individual reader (about $25 for a 24-hour access or via paid individual subscriptions or subscriptions of academic institutions or libraries for affiliated researchers and practitioners). While still a minority business model of scientific publishing, the shift of costs to the author or author's funding source has grown and been shown financially viable. The Wall Street Journal listed "open access" as among the ten most important medical news events of 2003. It has only increased in importance since.

At the moment, only about 10% of published scientific articles are accessible without restrictions. In response to the advantages to scientists and the almost prohibitive costs to libraries for serial subscriptions, major research foundations such as the UK Research Councils, the Howard Hughes Medical Institute and the Welcome Trust have established policies that research they support be deposited in publicly available, open access repositories like PubMed Central. This is also the policy of the US NIH, although it is on a voluntary basis. All articles published in *Environmental Health *are deposited automatically and immediately in PubMed Central, where they remain permanently, regardless of what happens to any individual journal or the publisher.

The easy accessibility of open access research articles retrieved via computer databases such as PubMed has allowed many journals to succeed where in earlier days they would have required subsidies from professional associations or had very small print runs and circulations. Major journals continue to be "browsed" at frequent intervals by large fractions of the scientific community, but specialist articles are now more frequently identified by online searching. By using certain key words in automated searches, each scientist is becoming the "editor" of his or her own select journal, with weekly lists of new publications. Open access publication is ideally suited to this new paradigm in the use of the huge and expanding corpus of worldwide scientific literature.

## Journal functions

Although publishing has a central role in the production and dissemination of scientific and technical knowledge, the basic design of scientific journals goes back to the 17^th ^century and has changed little in format, even in the age of electronic publishing. This doesn't mean that electronic distribution and storage hasn't made some important differences, however. In addition to the speed and low cost of distribution, the ability to use digital rather than physical storage has meant that journals no longer must choose among meritorious manuscripts because of page constraints. Articles can also be longer, have supplemental material like data files of use to specialist researchers, extended and detailed tables and even multimedia presentations. Color and animations now come at no extra cost. So while the wrapping of the package looks much the same, the contents are fuller, richer and more varied.

Indeed, the US National Research Council (2001) highlighted the potential for a shift from original publications to original data: "the expanding availability of primary sources of data in digital form may be shifting the balance of research away from working with secondary sources such as scholarly publications..."[2]. At the same time, some funding agencies, especially the US National Institutes of Health, are requiring that original data are shared after the researchers themselves have had the initial opportunity to publish their analysis and conclusions. The Internet offers a unique chance of linking publications to repositories of primary data.

*Environmental Health *offers the opportunity to upload 'additional files', which will be made available along with the published article. They can include raw materials, original documentation, and software. Several authors have already chosen to take advantage of this opportunity (to facilitate downloading, each file is limited to 10 megabytes). When linked to a published article, these additional files serve as a repository of original data for other scholars to use after appropriate credit (as required by the Open Access charter and the accompanying license)[1].

## Peer review

New technologies and opportunities must continue to respect practices and policies crucial to ensuring the quality and validity of the research. In particular, this means retaining the value and accountability provided by peer review. As a key to quality assurance, scientific manuscripts are assessed by peers and revised accordingly before publication. Review procedures differ between journals, and some have experimented with different forms, although no single model has been found to be ideal.

Our editorial board has played a key role in securing a high quality standard of *Environmental Health*. We aim at having one editorial board member assess the merits of each manuscript. In response to the increased flow of manuscripts, we have now expanded the editorial board. In addition, since some manuscripts require review of the statistical methods, we have established a board of statisticians. The list of board members can be retrieved from the *Environmental Health *website [3].

One of the main issues regarding peer review is whether the author or reviewer, or both, should be anonymous. *Environmental Health *has adopted open peer review: authors and reviewers know one another's identity. In addition, the reviews are available from the "Prepublication history" record at the left-hand panel with the abstract or full-text version of all published articles. This option makes it possible for readers to appreciate the original submission along with comments from identified reviewers, with the authors' responses.

Open and published peer review serves two major purposes. We believe the effort of our hard-working reviewers deserves credit, and we hope that publication of their opinion may provide the appropriate recognition. We also believe transparency is a must in environmental health, and readers have a right to know why a particular article was published and why revision was requested.

*Environmental Health's *open review policy, while a departure from much past practice, has shown itself able to produce unusually helpful and constructive reviews, something noted by both reviewers and authors. For those of you who have helped us as reviewers we want to take this opportunity to express our gratitude once again for the great service you have done the journal, its authors and, most importantly, the profession. We also note that the great majority of our colleagues have embraced open peer review, and we have received very few negative reactions from prospective reviewers who insist on being anonymous.

Readers also play a role in securing transparency and quality by commenting on published articles, as can be easily done via the "Post a comment" at the left-hand panel when retrieving an abstract or full-text version. In *Environmental Health*, accepted comments will be immediately available to the readers. As one of our colleagues once remarked, *real *peer review begins after publication. We invite all our readers to participate in the post publication evaluation of the literature through our comment facility.

## Conflicts of interest

A conflict of interest policy specifies the ethical considerations relevant to ensuring impartiality. Ideally, academic writing should be objective, impersonal and informational. But no academic researcher can consider him/herself completely free of all influences from vested interests. Undue influence from vested interests can easily affect the conduct of science and its conclusions [4]. Recent reviews of environmental health publications have shown that studies supported by industry sources are more likely to be favorable to industry interests [5,6]. Although the reasons for these discrepancies are no doubt complex, collaboration with interest groups can also be an advantage by facilitating the translation of the research into practical applications and by allowing access to important proprietary information. In regard to conflicts of interest, *Environmental Health *adheres to the policy of the International Committee of Medical Journal Editors (ICMJE) [7].

Many biomedical journals now require rather specific declarations of competing interests that might affect an author's objectivity [8]. In circumstances in which a significant conflict of interest exists,*Environmental Health *editors and reviewers will recuse themselves from evaluating manuscripts, and any additional issues must be declared. In the field of environmental health science, where social merit is crucial and effects on economic interests common, such declarations are particularly necessary. Even so, such declarations may not provide full insight, when important competing interests are left out [9]. Affiliation with, or support from, an industrial corporation or an environmental organization does not invalidate research, but transparency would demand the reader has access to those relationships as one more data point in judging the reported findings and conclusions. The same demand in regard to declaration of interests applies to us as editors, and our reviewers.

Publishing is also an economic activity of its own. The core scientific publishing market is a multibillion dollar business. Journal publishers therefore have commercial interests and need to attract authors, readers, advertisers and other sources of support [10]. The recent past includes incidents where editors of major biomedical journals were fired by publishers or resigned due to differences in opinion regarding journal content.

The Editors of *Environmental Health *are unpaid and independent of commercial issues. Waivers to publish free of charge or at a reduced rate in *Environmental Health *can be granted to authors in case of demonstrated need, e.g., in submissions from developing countries, but this decision now rests with the publisher and not the Editors. The Editors also have the right to screen advertisements relating to journal topic before they are posted on the website.

Differences of opinion may occur, and editorial decisions should be open to possible appeal. The Committee on Publication Ethics (COPE) [11] code requires that the journal has a mechanism for handling complaints, which may result in publishing corrections, apologies, and retractions. We have therefore established the position of Ombudsman, who is completely independent of the publisher and the editors. The Ombudsman is Dr. Anthony Robbins, himself a journal editor and distinguished public health professional, with wide experience in government and academia. The ombudsman is now listed with the editorial board.

## Ranking of journal influence

Both the quality and quantity of submitted manuscripts have risen and we are gratified by the attention many of our papers have received in the media and the profession. The most popular articles have been accessed (abstract or full paper) over 10,000 times via the journal website [12], plus likely also as many times via PubMed Central. Typically, a new article is downloaded several hundred times during the first month after publication. The record number of accesses for an article published by BioMed Central is approximately 100,000 [13], and for a specialist field like environmental health, the access numbers appear more than satisfactory.

Ranking of scientific journals usually relies on the so-called impact factor, reflecting the number of times articles published during two preceding years have been cited during the following year. The number of citations that an article receives over time will of course not be apparent immediately, but the total number of downloads during the first six months after publication predicts the number of citations after two years [14]. Evidence also indicates that open access, by itself, increases the number of citations [15].

We recently received the welcome news that Thomson Scientific (ISI) has decided to track *Environmental Health*. ISI Tracking is reserved for well established scientific journals and inclusion of the journal in Science Citation Index Expanded (available through the Web of Science) is confirmation of our success. As part of that tracking process we will receive an official impact factor in 2010. Using the ISI procedures, BioMed Central has computed a tentative, unofficial impact factor (based on citations of articles published in 2005 and 2006), a very flattering 2.10, placing *Environmental Health *among the most important journals in the field of environmental health science, despite its youth. We believe the official factor will only increase this further.

## The future

The experience of working with *Environmental Health*, its authors, reviewers, and readers, has been rewarding, eye-opening and gratifying. We are now convinced the choices we made five years ago in regard to open access and open peer review were the right ones. *Environmental Health *will continue to contribute fast delivery of environmental health science at high quality with easy and free access for readers. We thank you all for making the journal a success as readers, reviewers and authors. We look forward to an even bigger presence in the near future and we welcome you along for what has already been a spectacular ride.

## Competing interests

The author(s) are editors-in-chief of *Environmental Health *and declare that they have otherwise no competing interests.

## Authors' contributions

PG and DO jointly prepared the manuscript. Both authors have read and approved the final manuscript.
